# Cardiometabolic Health in Adolescents and Young Adults with Congenital Adrenal Hyperplasia

**DOI:** 10.3390/medicina58040500

**Published:** 2022-03-30

**Authors:** Ruta Navardauskaite, Kristina Semeniene, Marius Sukys, Agne Pridotkaite, Aurika Vanckaviciene, Birute Zilaitiene, Rasa Verkauskiene

**Affiliations:** 1 Department of Endocrinology, Medical Academy, Lithuanian University of Health Sciences, LT-50161 Kaunas, Lithuania; semeniene.kristina@gmail.com (K.S.); agne.pridotkaite@stud.lsmu.lt (A.P.); 2 Department of Genetics and Molecular Medicine, Medical Academy, Lithuanian University of Health Sciences, LT-50161 Kaunas, Lithuania; marius.sukys@lsmu.lt; 3 Department of Nursing, Medical Academy, Lithuanian University of Health Sciences, LT-50161 Kaunas, Lithuania; aurika.vanckaviciene@lsmu.lt; 4 Insitute of Endocrinology, Medical Academy, Lithuanian University of Health Sciences, LT-50161 Kaunas, Lithuania; birute.zilaitiene@lsmuni.lt (B.Z.); rasa.verkauskiene@lsmuni.lt (R.V.)

**Keywords:** CAH, congenital adrenal hyperplasia, cardiometabolic, insulin resistance, genotype and phenotype correlation

## Abstract

*Background and objectives:* Data on long-term cardiometabolic consequences in patients with congenital adrenal hyperplasia (CAH) are controversial. The aim of our study was to evaluate body mass index (BMI), body composition, blood pressure (BP) and insulin sensitivity in adolescents and young adults with CAH in comparison with healthy controls. *Methods:* Thirty-two patients with classical CAH (13 males; mean of age 26.0 ± 7.1, years (14.0–37.3) were compared to 32 healthy sex and age-matched controls (13 males; mean of age 28.7 ± 4.6 years (14.1–37.2), *p* = 0.13). Body composition was evaluated in all subjects with DXA (Hologic Inc., Bedford, MA, USA). Elevated BP was defined as BP > 95th percentile in adolescents, and >140/90 mmHg in adults. Comparisons between the two groups were adjusted for age, gender, pubertal stage and height. An oral glucose tolerance test was performed, and fasting insulin levels were evaluated. Insulin sensitivity was determined using a homeostasis model assessment of insulin resistance index (HOMA-IR). *Results:* The median BMI was significantly higher in subjects with CAH (1.63 (0.3–2.4) SDS and 0.41 (−0.63–1.19) SDS, respectively, *p* < 0.001). Visceral adipose tissue (VAT) in grams was significantly higher in CAH females versus control females (467 (231–561) vs. 226 (164–295), *p* = 0.002). Elevated BP was identified in 34% of CAH patients (nine SW and two SV) and 12.5% (*n* = 4) of controls (*p* = 0.038). Impaired fasting glycemia was detected in one SW CAH patient and impaired glucose tolerance in three SV CAH patients; normal glucose tolerance was found in all controls. A strong positive correlation was found between median cumulative hydrocortisone (HC) dose equivalents and LDL-cholesterol and a negative association with lean body mass (*r* = −0.79, *p* = 0.036) in females with CAH. BMI, VAT, BP and HOMA-IR were not related to median cumulative HC dose equivalents. *Conclusions:* CAH patients had higher BMI, VAT and frequency of elevated BP compared to controls. Doses of glucocorticoids were related directly to LDL-cholesterol and inversely to lean body mass in CAH females, but not associated with body composition, insulin sensitivity and BP in the whole cohort of CAH patients.

## 1. Introduction

Congenital adrenal hyperplasia (CAH) is an autosomal recessive disorder that results from impaired steroidogenesis in the adrenal cortex. The estimated prevalence of CAH is 1:10,000 and annual incidence ranges from 1:5000 to 1:20,000 [1,2]. In 95% of cases, CAH is caused by mutation of the *CYP21A2* gene that encodes the enzyme 21-hydroxylase (21OH) [3,4]. Due to a deficiency of 21OH (21OHD), the synthesis of cortisol and aldosterone is impaired. The CAH treatment aims to replace glucocorticoid (GC) and mineralocorticoid (MC) to prevent adrenal insufficiency and androgen excess. Dose adjustments are based on clinical symptoms and adrenal androgens and steroid precursor levels, such as 17-hydroxyprogesterone (17OHP), androstenedione (4-A), and testosterone (T) [5]. The therapeutic doses of GC treatment are supraphysiological, and the general average maintenance dosage varies between 9 and 26.5 mg/m^2^/day of hydrocortisone (HC) divided into 2–3 doses [6]; however, the current guidelines from the Endocrine Society recommend, in growing individuals with classic CAH replacement therapy, HC at 10–15 mg/m^2^/day [7]. In the management of patients with CAH, it is a challenge to find the right balance of GC doses to suppress adrenal androgen hypersecretion and avoid hypercortisolism which, among other side effects, are associated with increased risk of cardiometabolic disorders [8]. 

Cardiometabolic disease describes a variety of conditions beginning with insulin resistance, progressing to metabolic syndrome, pre-diabetes, and finally to more severe conditions including cardiovascular disease (CVD) and type 2 diabetes (T2DM). These conditions are grouped under the umbrella term “cardiometabolic disease” as they are related or share risk factors, such as overweight and obesity, dyslipidemia, and high blood pressure (BP) [6,9,10]. 

Several studies have reported an increased prevalence of obesity, as well as increased insulin resistance and BP levels in CAH patients. Body composition and fat distribution are also compromised in CAH patients [11,12,13]. However, data on the association of cardiometabolic risk factors and clinical form as well as GC doses in CAH youth are controversial. Moreover, different variants in the *CYP21A2* gene mutations can lead to a variable degree of loss of 21OHD activity, which can result in various clinical presentations.

In this study, we aimed to evaluate cardio-metabolic risk factors in transition-age adolescents and young adults with classical CAH with different residual 21OHP function, treated in a single university centre, and compare with age- and sex-matched healthy controls.

## 2. Materials and Methods

### 2.1. Subjects

**Figure 1:**
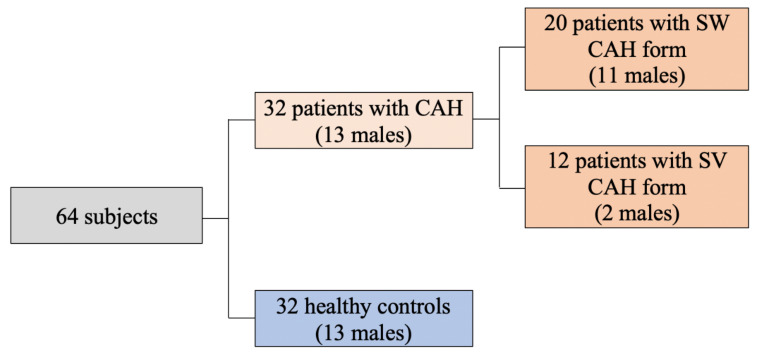
Descriptive scheme of study subjects.

All patients older than 14 years from the Lithuanian database of CAH patients were invited to participate in the study. All patients of the study were born before the Newborn Screening Program for CAH establishment in Lithuania [14]. Thirty-two adolescents and young adults (14–37 years of age) with CAH were recruited and compared to 32 healthy control subjects rigorously matched for age, sex and ethnicity (Figure 1). Eight patients in both the CAH and control groups were adolescents (14–18 years old). Puberty was evaluated according to the Tanner stage. In each study group, one patient (12.5%) was in Tanner stage 3, three (37.5%)-in Tanner stage 4, and four (50%)-in Tanner stage 5. 

### 2.2. Biochemical and Molecular Diagnosis of CAH

The diagnosis of 21OHD was confirmed by the mutation analysis of the *CYP21A2* gene. 

All patients were genotyped for confirmation of the diagnosis. *CYP21A2* gene and *CYP21A1P* pseudogene copy number analysis was performed using quantitative multiplex ligation-dependent probe amplification (MLPA) with SALSA^®^ MLPA^®^ probemix P050-C1 CAH (MRC-Holland, Amsterdam, The Netherlands); reference sample—SD039-S02 Reference DNA (MRC-Holland). The detection of the sequence changes of the *CYP21A2* gene was performed using Sanger sequencing after selective long-range PCR with primers specific for *CYP21A2* and/or *CYP21A1P* [15].

Twenty (60%) patients had the salt wasting (SW) form of CAH, and 12 patients (40%) has the simple virilising (SV) form. The definition of CAH forms was based on the *CYP21A2* genotype, initial and follow-up plasma renin concentrations, and electrolyte status at diagnosis: severe *CYP21A2* mutations on both alleles, or complete gene deletions/conversions and significantly elevated renin concentrations or apparent salt loss at the time of diagnosis of CAH were indicative of the SW form of CAH. Patients with severe *CYP21A2* mutations on one allele and mild on another allele causing mild 21OHD with normal or only slightly elevated renin concentrations and normal electrolyte levels were diagnosed with a simple virilizing form of CAH.

Stratification of common *CYP21A2* pathogenic variants by residual enzyme activity was based on Krone et al. and Concolino et al. [16,17], and is detailed in Table 1.

Genotypes were classified according to residual 21OH activity (Null, A, B, C). For further analysis, CAH patients were divided into 2 subgroups according to genotype: the 1st subgroup (*n* = 17) included patients with mutations that caused 0% or close to 0% of 21OH activity (Null mutations in both alleles or Null in one and A type mutation in another allele); the 2nd subgroup (*n* = 15) included other combination of mutations, associated with ≥1% of residual 21OH activity.

### 2.3. Therapy

GC doses are expressed as the median cumulative dose per body surface (milligrams per square meter per day). The median dose of GC was calculated as the actual cumulative corticosteroid dose during all treatment periods. All the GC doses used were converted into hydrocortisone (HC) dose equivalents for the purpose of normalization using anti-inflammatory equivalents (20 mg of hydrocortisone = 5 mg of prednisolone (PD) = 0.75 mg of dexamethasone (DEX)) [18]. Five patients (15.6%) were treated with PD, and 6 (18.8%) with DEX; the rest were treated with HC. 

Treatment efficacy was assessed in at least 2 of 4 annual measurements of serum 17OHP (12–32 nmol/L), T (T levels were evaluated according to the chronological age and sex), and ACTH (normal range 1.63–14.15 pmol/L) measurements. MC replacement was monitored by blood pressure and renin concentration (normal range 1.6–14.7 ng/L), sodium level (normal range 136–146 mmol/L) and potassium level (normal range 3.5–5.1 mmol/L).

### 2.4. Physical Examination

Anthropometric measurements of height (centimetres) and weight (kilograms) were performed in all participants. Height was measured using a Harpenden stadiometer. Height standard deviation score (Ht-SDS) was calculated according to age and gender using Lithuanian National Children Growth Evaluation Chart references for all study subjects under 18 years [19]. Waist circumference (WC) and hip circumference were measured by two investigators (RN and KS). Abnormal WC was defined according to the Lithuanian National Children Growth Evaluation Chart assessment criteria if above the 90th percentile in subjects younger than 18 years old [19]: above 102 cm in adult males and 88 cm in adult females [20]. Waist to hip ratio (WHR) was measured as the ratio between the circumferences of the waist (the narrowest part of the torso between the 12th rib and the iliac crest) and hip (the maximal extension of the buttocks). Normal WHR was defined if less than 0.9 for adult males, and less than 0.85 for adult females. Body mass index (BMI) standard deviation score (SDS) was calculated using Lithuanian National Children Growth Evaluation Chart references [19]. A BMI between 25 and 29.9 kg/m^2^ (>+1 SD and <+2 SD) was classified as overweight, a BMI of 30 kg/m^2^ or more (>+2 SD) as obesity. Waist to height ratio (WHtR) is emerging as an important measure of abdominal obesity, with a strong predictive value for metabolic syndrome and CVD risk. An abnormal WHtR was defined if above 0.5 for children and adults [21,22]. The average of three BP measurements taken with a sphygmomanometer was used for the analyses. Elevated systolic or diastolic BP was defined as a value above the 95th percentile for age and sex (adjusted for height) in adolescents [23], and higher than 140/90 mmHg in adults.

### 2.5. Laboratory Investigations

To evaluate the metabolic profile, a blood sample from each study subject was collected after at least 8 h of fasting before GC and fludrocortisone administration, at 8:00–9:00 am from the antecubital vein catheter for measurements of fasting glucose, insulin, total cholesterol (total-C), high-density lipoprotein cholesterol (HDL-C), low-density lipoprotein cholesterol (LDL-C) and triglyceride (TG) levels. Increased TG levels were characterized as values ≥1.95 mmol/L. An HDL-C level ≤ 1.55 mmol/L, LDL-C > 2.59 mmol/L, and total cholesterol > 5.5 mmol/L were considered abnormal, and a fasting glucose ≥ 5.6 mmol/L was considered as an impaired fasting glycemia [24]. 

The oral glucose tolerance test (OGTT) was performed: a fasting blood sample was obtained at time 0 (between 08.00 and 09.00 h) for the measurement and another sample of glycemia was obtained 120 min after the oral administration of 1.75 g/kg (maximum 75 g) of glucose. Glucose tolerance was evaluated using the criteria of the World Health Organization [25]. Insulin resistance (IR) was estimated using the homeostasis model assessment (HOMA) method according to the formula: IR = [insulin (mU/L) × glucose (mmol/L)]/22.5 [26]. Elevated HOMA-IR index was defined as above 2.5 in adults and above 3.16 in adolescents [27].

### 2.6. Body Composition Assessment

Whole-body dual-energy X-ray absorptiometry (DXA; Hologic, Marlborough, MA, USA) was used to measure total fat mass (TFM) in grams and Z-score, total percent fat, visceral abdominal tissue (VAT) mass, and total lean body mass. Subcutaneous adiposity tissue (SAT) was calculated as follows: SAT = total body fat mass–VAT [28].

### 2.7. Biochemical and Hormonal Assays

Concentrations of testosterone (T, nmol/L; Biosource, Nivelles, Belgium), 17-hydroxyprogesterone (17OHP, nmol/L; DIAsorource, Louvain-la-Neuve, Belgium), sex-hormone-binding globulin (SHBG, nmol/L; Immunotech, Prague, Czech Republic), adrenocorticotropic hormone (ACTH, pmol/L; DIAsorource, Louvain-la-Neuve, Belgium), renin (ng/L; DIAsorource, Louvain-la-Neuve, Belgium), insulin (mU/L; DIAsorource, Louvain-la-Neuve, Belgium), sodium (Na, mmol/L; Beckman Coulter, Prague, Czech Republic, and potassium (K, mmol/L; Beckman Coulter, Prague, Czech Republic) were measured by immunoradiometric assay. 

### 2.8. Diagnostic Criteria for Metabolic Syndrome

Metabolic syndrome (MetS) was defined using the modified National Cholesterol Education Program Adult Treatment Panel III devised by the American Heart Association and National Heart, Lung, and Blood Institute [29]. MetS was diagnosed if adults had any 3 of the following: fasting glycemia ≥ 5.6 mmol/L, BP ≥ 130/85, HDL-C < 1.29 mmol/L (females), and <1.03 mmo/L (males), TG ≥ 1.7 mmol/L, or waist circumference (WC) ≥ 102 cm (men) or ≥88 cm (women). For pediatric patients under 18 years, MetS was defined using Weiss et al. criteria [30] if they had 3 or more of the following: fasting glycemia ≥ 5.6 mmol/L, BP > 95th percentile, TG > 95th percentile, HDL < 5th percentile, or BMI ≥ 95th percentile.

### 2.9. Statistics

Statistical analyses were performed using SPSS 27.0 software (SPSS Inc., Chicago, IL, USA). Continuous variables are given as median and interquartile range and means and standard deviations (M ± SD). As the analyzed values were non-normally distributed, the Mann–Whitney U test was used for comparisons and Spearman’s correlation coefficient was calculated to evaluate the correlation of continuous variables. The differences were considered statistically significant when *p* < 0.05.

## 3. Results

### 3.1. Cardiometabolic Risk Factors

Clinical characteristics of patients with CAH and matched controls are shown in Table 2.

#### 3.1.1. BMI and Adipose Tissue Distribution

**Figure 2:**
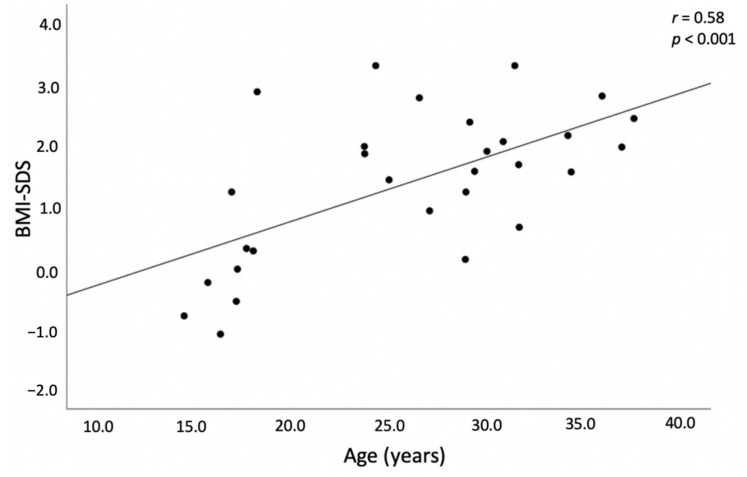
Relationship between age and BMI (SDS) in patients with CAH.

Mean BMI-SDS was significantly higher in subjects with CAH than healthy controls (*p* = 0.002) and in males with the SV form compared to those with the SW form (*p* = 0.009). Overweight (BMI > 1.0 SDS) was identified in 36.6% of CAH patients vs. 31% of controls: *p* = 0.64 and obesity (BMI > 2.0 SDS) in 30 vs. 0%, respectively, in adults. A strong positive correlation between BMI-SDS and age was found (*r* = 0.58, *p* < 0.001; Figure 2).

WHR was significantly higher in CAH patients compared to controls and in SV compared to SW males’ group. TFM, VAT and SAT mass were significantly higher in males in the SV group than in males in the SW group, and in the total CAH female group than in the control female group (*p* = 0.002). An abnormal WHtR was found in 50% of patients with CAH and in 10% of controls, *p* < 0.001.

#### 3.1.2. Blood Pressure

Elevated BP was identified in 34% of CAH patients, and in 12.5% of controls, *p* = 0.038. Higher BP was recorded in 45% (*n* = 9) with SW and in 16.6% (*n* = 2) with SV CAH (*p* = 0.1). Comparing CAH patients with elevated and normal BP, no significant differences were found in age, adiposity indices, HC and MC doses, insulin sensitivity, hormone levels, or electrolytes.

#### 3.1.3. Glucose Metabolism and Insulin Resistance

CAH patients had significantly lower fasting glycemia compared to controls (*p* = 0.009). Fasting insulin concentration was significantly higher in the CAH group compared to control females (*p* = 0.001). HOMA-IR was elevated in 62.5% (*n* = 20) of CAH patients and in 18.75% (*n* = 6) of control subjects, *p* < 0.001. Impaired fasting glycemia was detected in one SW CAH patient (30 years old), and impaired glucose tolerance in three SV CAH patients (two males of 29 years old, and one female of 30 years old), and normal glucose tolerance was found in all controls.

#### 3.1.4. Dyslipidemia

Total-C was within the normal range in both CAH groups, but was higher in the SV vs. SW group (*p* = 0.046). HDL-C was significantly lower in the SW group than in the SV group (*p* = 0.023). 

### 3.2. Hormonal Profile

An unsuppressed (>32 nmol/L) 17OHP was observed in 18 CAH patients (56%). Median T concentration was higher in CAH compared to the control females (*p* = 0.05). Median ACTH levels in the SW and SV CAH groups did not significantly differ (92.2 ± 1.44.3 and 29.0 ± 60.0, *p* = 0.18).

In the CAH patients, T levels were significantly negatively related to fasting insulin concentration (*r* = −0.47, *p* = 0.047), and a strong direct relationship was evident between ACTH and 17OHP levels (*r* = 0.66, *p* = 0.001).

### 3.3. Relationship of HC Doses with Body Composition, Insulin Sensitivity, Blood Pressure and Lipid Profile

**Figure 3:**
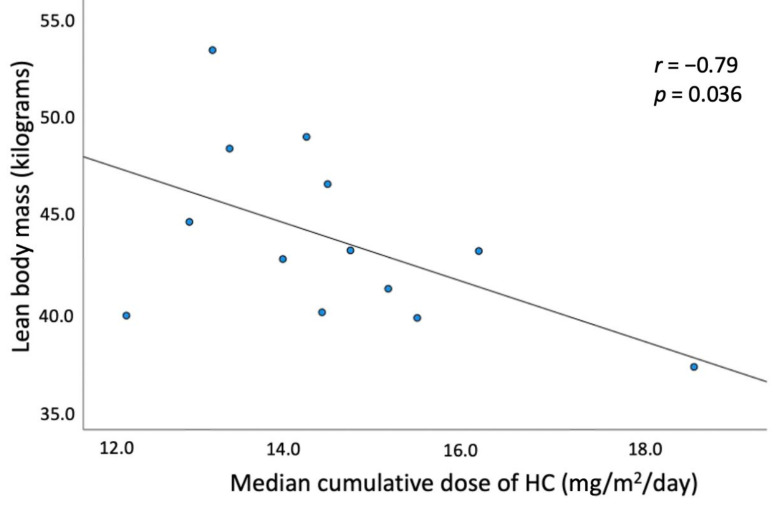
Negative association between HC dose (mg/m^2^/day) and lean body mass (kilograms) in CAH female group.

There was no significant correlation between the cumulative median dose of HC equivalent and BMI-SDS, VAT, SAT, TFM (in grams, and Z-score), VAT/SAT ratio, WHR, WHtR, lipids, fasting glycemia and glycemia post-OGTT, insulin levels, and HOMA-IR index in patients with classic CAH. A strong negative correlation was found between the cumulative median dose of HC equivalent and body lean mass in grams in females with CAH (*r* = −0.79, *p* = 0.036) (Figure 3).

Analyzed in patients’ subgroups according to GC type used (HC, PD, DEX), no significant differences between the cumulative median dose of HC equivalent and BMI-SDS, VAT mass in grams, SAT, VAT/SAT ratio, WHR, WHtR, lipids, fasting glycemia and glycemia post-OGTT, insulin levels, or HOMA-IR index were found.

The median HC doses did not significantly differ between groups with elevated and normal BP (15.7 versus 14.9 mg/m^2^/d, *p* = 0.34). Systolic and diastolic BP adjusted for age and Ht-SDS did not significantly correlate with either cumulative median HC dose equivalent (*r* = 0.632, *p* = 0.25 and *r* = 0.28, *p* = 0.63, respectively), or with cumulative median MC dose (*r* = 0.32, *p* = 0.59 and *r* = 0.58, *p* = 0.31, respectively).

A strong positive correlation was detected between HC doses and LDL-C in females with CAH (*r* = 0.636, *p* = 0.048), and TG in males with CAH (*r* = 0.786, *p* = 0.036). 

### 3.4. Metabolic Syndrome

None of study participants had three or more pathological findings relating to MetS criteria, and MetS was not confirmed.

### 3.5. Genotype and Phenotype Correlation

Severe genotypes (null and A) demonstrated a good correlation with the expected phenotype, with positive predictive values (PPV) of 100 and 85.7%, respectively, whereas the less severe genotype B demonstrated a weaker correlation with a PPV of 57.1%. Patients with genotype Null in both alleles needed a significantly higher HC dose in infancy compared with patients with other combinations of mutations (21.4 (19.3–28.7) and 18.4 (16.6–19.3) mg/m^2^/d, respectively, *p* = 0.05).

Patients with Null/Null and Null/A genotypes had significantly lower BMI-SDS (*p* = 0.004), total-C (*p* = 0.046), LDL-C (*p* = 0.004) and TFM in kg (*p* = 0.041), compared to patients with other types of mutations with higher residual 201OHP activity (Table 3).

## 4. Discussion

In this study, we have described the higher risk of cardiometabolic consequences in CAH patients, such as obesity, insulin resistance and hypertension. In the literature, data on the long-term cardiovascular and metabolic outcomes in CAH patients are rather controversial. 

### 4.1. BMI and Adipose Tissue Distribution

Increased BMI and unfavorable body composition have been described in several cohort studies with the classical form of CAH patients. In one of the largest cross-sectional studies with 165 adults conducted in the United Kingdom (CaHASE), obesity and overweight were found in 41 and 37% of CAH patients; however, suppressed 17OHP levels were found in almost half of the patients of both genders, suggesting overtreatment might contribute to overweight and obesity [10]. In our study, overweight and obesity were present in 37 and 30%, respectively; moreover, in contrasting the study cited above, 17OHP levels in most patients of our cohort were above the recommended range, possibly to some degree reflecting non-compliance, since median cumulative GC doses were also rather elevated as compared to that recommended in the recent guidelines [7]. In some [31,32,33] but not all [34,35,36] studies in children, adolescents and young adults, total cumulative GC doses were determinant of BMI. In our study, neither BMI, nor peripheral (SAT) or visceral (VAT) adiposity indices were associated with median cumulative GC doses in our patients. 

Body fat distribution seems also to be altered in CAH patients. In our study, CAH females had significantly higher VAT and there was a trend to higher VAT in the whole CAH cohort compared to controls. Consequently, VAT/SAT ratio was also significantly higher in CAH patients. Similarly, in the study by Kim et al., the VAT/SAT ratio was increased in young adult CAH patients compared to BMI-matched controls, indicating metabolically unfavorable fat distribution for the same degree of adiposity [11].

Interestingly, patients with the SV form of CAH had higher BMI-SDS, total fat mass, VAT and SAT compared to the SW patients’ group, although the median of total cumulative GC doses were similar in both groups. Moreover, due to later diagnosis, SV patients were exposed to GC treatment for a shorter time; therefore, GC therapy does not seem to be a major determinant of the development of obesity in these patients. Furthermore, VAT/SAT ratio was similar, indicating a comparable fat distribution pattern in both groups. It has been suggested that, in addition to GC type and dose, genetic factors responsible for GC metabolism and receptor sensitivity might be involved in the predisposition to adverse cardiometabolic outcomes. The glucocorticoid receptor (GR) consists of two alternatively spliced isoforms: GRα, which activates gene transcription, and GRβ, a dominant-negative receptor. These different actions of GR could explain the low impact of the GC dose on cardiometabolic complication development. Two variants of the dominant-negative GRβ, in conjunction with a common *Bcl1* intron variant, resulted in hypersensitivity to endogenous and exogenous glucocorticoids. CAH patients with the *Bcl1* variant of the GC receptor gene—which increases the receptor’s activity—could have higher BMI, waist circumference, and systolic blood pressure [37], while GRα works oppositely and decreases GR activity, and could be related to a lower risk of development cardiometabolic complications, regardless of higher GC dose [38].

Studies in children with CAH indicate that overweight and obesity are already present in childhood. The BMI of normally growing children with CAH has been found to increase throughout childhood more than the expected age-related increase [13]. According to the reports from German and USA studies, the risk of obesity was increased in children with obese parents [32,36]. Although we did not analyze the parents’ BMI, in our study cohort CAH patients’ BMI increased with age and a significantly higher BMI was observed in adults than in children; therefore, it might be speculated that parental obesity is not a major determinant of CAH patients BMI.

### 4.2. Glucose Metabolism and Insulin Resistance

CAH patients in our study had significantly lower fasting glycemia compared to controls. It has been hypothesized that lower fasting glucose in CAH patients might reflect relative glucocorticoid and/or epinephrine deficiency, which has been shown to be implicated in glucose homeostasis during exercise in CAH patients [13]. However, CAH patients had higher fasting insulin and HOMA-IR—data consistent with a previous report [13]. In a recent systematic review that included 300 CAH children and adolescents and 137 adults with CAH, fasting glucose and insulin levels, glucose, and insulin after 2 h post-glucose load were comparable to control subjects; however, HOMA-IR was significantly higher in CAH patients [6], and thereby consistent with our study results.

Insulin resistance was directly related to VAT mass and negatively associated with lean mass in CAH patients. Lee et al. demonstrated that decreased skeletal muscle mass was associated with dyslipidemia, regardless of the presence of abdominal obesity, and suggested that insulin resistance may be associated with low muscle mass [39].

Higher androgen concentrations, hypercortisolism and unfavorable body composition may contribute to decreased insulin sensitivity in CAH patients [31]. In the present study, insulin levels were significantly directly related to T concentration, but no association of HOMA-IR with HC dose equivalents was found. Similarly, Sartorato et al. did not find any correlation between HOMA-IR index and cumulative HC doses [40]. In contrast, the study by Torky et al. reported suppressed T levels being significantly associated with insulin resistance, possibly indicating the effect of higher doses of GC [36].

Although insulin resistance was documented in most studies on glucose homeostasis in CAH patients, there were no reports on increased diabetes prevalence, except in a recent publication from the data of a Swedish registry of CAH patients where a three-fold higher prevalence of diabetes among CAH patients was reported; unfortunately, data on glucocorticoid doses were not available in this study [9]. In our study, 2-h post OGTT, glucose levels were significantly higher in CAH patients, and three adult patients (9%) had impaired glucose tolerance compared to none in the control group, indicating the need for further glucose metabolism monitoring in these patients.

### 4.3. Blood Pressure

Elevated blood pressure was identified in one-third of the CAH patients and was significantly more frequent compared to controls.

Data on the prevalence of hypertension among CAH patients are conflicting. Interestingly, according to the report from a study carried out in Germany and the USA, the prevalence of hypertension was higher in younger children than in adolescents (18.5% vs. 4.9%) [11,36]. Until 8 years of age, fludrocortisone dose/m^2^/day correlated significantly with BP in a regression analysis [41]. In our study, increased BP was evidenced in patients of different ages (from 14 to 37 years old). Although there were no differences in treatment, hormonal or cardiometabolic parameters between patients with elevated or normal blood pressure, hypertension was detected in almost half of CAH patients with the SW form, compared to only 12% in the SV group, possibly indicating the impact of MC use on blood pressure in CAH patients. This association was reported in a recent study in USA [36].

In a recent study from Egypt, slightly higher systolic and diastolic BP were found in children treated with prednisolone than in those treated with HC [28]. In our study, all CAH patients were treated with HC until the achievement of adult height, and there was no difference in BP between groups of patients later treated with different forms of GC.

According to previous studies, the first wave of hypertension can be developed in early infancy or childhood, which is associated with physiological MC resistance in infants and young children [36,41]. The second wave of hypertension development is associated with ageing and cumulative cardiovascular risk factors, as we identified in our study and other cohort studies with long-term follow-up data [9,10,36,42].

### 4.4. Dyslipidemia

CAH patients in our study had total cholesterol, LDL-C, HDL-C and TG within the normal range. In the subgoups analysis, total cholesterol was higher in the SV versus SW group, which might be explained by the higher BMI in SV patients. However, HDL-C was was also significantly higher in SV compared to SW patients. Studies on lipid profile in CAH patients presented controversial results. In the recent systematic study with a large number of CAH patients cited above, no significant differences in total cholesterol, its fractions and triglycerides, were found compared to controls [6]. Conversely, in the CaHASE study, as much as 46% of patients had hypertrigliceridemia and 39% had increased LDL-C levels (10). Accordingly, the national registry of CAH patients in Sweden reported a high frequency of dyslipidemia, most prominent among men with the SW form with Null genotypes [9]. On the contrary, data from pediatric CAH cohort studies indicated that most CAH patients had TG and LDL-C lower than the 50^th^ percentile. Similar to our study, lipid profile was influenced by the clinical form of the disease in the study by Moreira et al. SW patients had higher TG and LDL-C levels and lower HDL-C levels compared to SV patients [34].

### 4.5. Genotype and Phenotype Correlation

In our study, severe genotypes (Null/Null and Null/A) demonstrated a good correlation with the expected phenotype, as in previous studies [42,43,44,45,46,47,48]. In contrast, in one of the largest studies with 1507 patients by New et al., a genotype–phenotype correlation was observed in less than 50% of cases [49]. This has been linked to high numbers of patients with the compound heterozygous genotype of Null or A type mutation in one allele, and C type (p.Pro31Leu, p.Val282Leu) in another allele, which causes a less severe form of CAH. Interestingly, CAH patients with severe mutations in the *CYP21A2* gene (Null/Null and Null/A genotypes) in our study were significantly leaner and had more favorable lipid profiles compared with less severe mutations, resulting in higher residual 21OHP activity. Our data are consistent with other studies analyzing genotype–phenotype associations, which have reported individuals with SV and non-classical forms of CAH as being most affected by cardiovascular and metabolic morbidity compared to more severe genotypes [9,42]. It has been suggested that delayed diagnosis and prolonged periods of hyperandrogenism might underly these associations. Moreover, in a large Swedish study, the frequency of diabetes was increased in females with SV and nonclassical forms of CAH, and it has been claimed that both hyperandrogenism and the need of higher doses of GC to suppress symptoms of hyperandrogenism in females could explain why females are more affected [9]. This data could confirm the relationship with the negative effect of a longer period of treatment and higher HC doses in the salt wasters group. Our study has shown controversial data with detected impaired fasting glycemia in only SW CAH patients, and impaired glucose tolerance in three SV CAH patients; we also endorse the correlation between obesity and higher risk of metabolic disease as found in Sweden‘s study.

In the Swedish study, hypertension occurred more often in females (OR, 4.1) and remained significant only for SV and I172N females (B type) [9]; although we did not find a relationship with HC doses, elevated BP was mostly found in the SW group and occurred mainly in males.

The Swedish researchers reported hyperlipidemia in the total cohort and in the SW and Null (males) subgroups; in our group the data was opposite, and dyslipidemia (higher LDL-C and TG) was observed in SV females [9].

The main limitation of this study was its small sample size, as in many studies of rare diseases. Moreover, it should be emphasized that we reported several cardiometabolic risk factors, but not actual diseases, since patients in our cohort were relatively young and therefore need further long-term follow-up. Optimization of the replacement therapy with modified-release forms of GC that mimic physiological cortisol secretion appear to reduce hyperandrogenism while avoiding hypercortisolemia, and thus might eventually result in better long-term body composition, counteracting the cardiometabolic risk factors identified in CAH patients.

## 5. Conclusions

Compared to age-matched healthy controls, CAH patients have a higher prevalence of cardiovascular and metabolic risk factors, the majority of which are not associated with cumulative GC doses, except for the direct association with LDL-cholesterol concentration, and the inverse association with lean body mass in CAH females. Regular long-term follow-up of CAH patients is needed, with the aim of preventing obesity and other cardiometabolic conditions, in addition to the close monitoring of GC doses, especially in female patients.

## Figures and Tables

**Table 1:** Stratification of common *CYP21A2* pathogenic variants by residual enzyme activity.

Enzyme Activity	Phenotype	*CYP21A2* Pathogenic Variant	Grouping of Mutations According to 21OH Activity
0%	Severe (classic)	Whole-gene deletion Large-gene conversion p.Gly111ValfsTer21 p.[Ile237Asn;Val238Glu;Met240Lys] p.Leu308PhefsTer6 p.Gln319Ter p.Arg357Trp	Null
<1%		c.293-13A > G c.293C > G	A
2–11%		p.Ile173Asn	B
~20–50%	Mild (non-classic)	p.Pro31Leu p.Val282Leu p.Pro454Ser	C

21OH: 21-hydroxylase.

**Table 2:** Clinical characteristics of the CAH patients and matched controls.

Variables	SW/M (*n* = 11)	SV/M (*n* = 2)	SW/F (*n* = 9)	SV/F (*n* = 9)	SW (*n* = 20)	SV (*n* = 12)	All CAH (*n* = 32)	Controls (*n* = 32)
Age (years)	26.6 (16.9–29.2)	30.2 (28.9–30.45)	24.3 (20.8–28.4)	30.9 (20.9–36.5)	25.0 (17.1–29.2) ^c^	30.9 (23.7–36.0) ^c^	26.9 (17.9–31.6)	28.6 (17.1–31.3)
Median cumulative HC dose (mg/m^2^/day)	16.6 (14.2–17.7)	13.5 (13.3–13.5)	16.8 (13.6–20.7)	14.4 (12.3–16.1)	16.6 (14.1–18.5) ^c^	13.9 (12.6–15.2) ^c^	15.4 (13.2–17.7)	
Median cumulative MC dose (μg/day) *	100 (87.5–125)		100 (50–100)		100 (50–125)			
BMI-SDS **	0.31 (−0.4–1.87)	2.28 (1.24–2.5)	1.84 (1.14–3.01) ^b^	1.8 (1.7–2.63) ^b^	0.93 (−0.03–2.17)	1.98 (1.57–2.82)	1.63 (0.3–2.4)	0.41 (−0.68–1.19)
WHR	0.79 (0.76–0.9)	1.0 (0.9–1.1)	0.86 (0.81–0.95) ^b^	0.84 (0.79–0.91) ^b^	0.85 (0.78–0.9)	0.88 (0.8–0.94)	0.85 (0.78–0.91) ^d^	0.79 (0.76–0.84) ^d^
WHtR	0.45 (0.41–0.52)	0.65 (0.55–0.7)	0.54 (0.47–0.61) ^b^	0.58 (0.53–0.59) ^b^	0.48 (0.41–0.55) ^c^	0.58 (0.54–0.6) ^c^	0.52 (0.44–0.58) ^d^	0.44 (0.4–0.47) ^d^
Systolic BP (mmHg)	129 (117–138)	127 (121–129)	130 (124–135) ^b^	120 (113–125) ^b^	130 (120–138) ^c^	121 (120–126) ^c^	125 (120–132)	122 (112–131)
Diastolic BP (mmHg)	82 (72–90)	80 (77–82)	78 (68–92)	80 (74–80)	82 (72–92)	80 (77–80)	80 (72–86)	80 (70–83.7)
Sodium (mmol/L)	138 (136–141)	136 (136–136)	139 (135.3–139)	137 (133.2–138.8)	138 (136–139)	136 (133.5–138.5)	138 (135.2–139)	139 (137–140)
Potassium (mmol/L)	3.8 (3.6–4.0)	3.9 (3.9–3.94)	3.8 (3.6–4.0)	3.9 (3.6–3.9)	3.8 (3.6–4.0)	3.7 (3.6–3.9)	3.8 (3.6–4.0)	4.0 (3.8–4.2)
Total-C (mmol/L)	3.84 (3.27–4.25)	4.5 (4.4–4.6)	4.63 (3.66–5.17)	4.42 (4.09–5.47)	3.98 (3.39–4.51) ^c^	4.46 (4.14–5.43) ^c^	4.2 (3.7–4.7)	4.5 (3.8–5.4)
HDL-C (mmol/L)	1.12 (0.98–1.38) ^a^	1.79 (1.57–1.85) ^a^	1.54 (1.38–1.54)	1.52 (1.36–1.71)	1.21 (1.01–1.58) ^c^	1.53 (1.39–1.74) ^c^	1.44 (1.12–1.64)	1.47 (1.27–156)
LDL-C (mmol/L)	2.18 (1.86–2.54)	2.64 (2.4–2.7)	2.62 (2.07–3.18)	2.75 (2.2–3.81)	2.27 (1.88–2.62)	2.72 (2.25–3.59)	2.49 (1.89–2.92)	2.69 (2.2–2.7)
TG (mmol/L)	1.01 (0.62–1.19)	0.8 (0.68–0.91)	1.36 (0.57–1.66)	1.24 (0.87–1.67)	1.07 (0.61–1.37	1.15 (0.78–1.65)	1.27 (0.72–1.66) ^d^	0.82 (0.57–1.01) ^d^
Fasting insulin (mU/L)	9.1 (3.5–12.3)	10.9 (9.8–12.3)	12.5 (10.1–14.0) ^b^	16.2 (8.6–23.0) ^b^	10.2 (5.1–12.9)	15.4 (9.2–22.3)	11.2 (7.8–20.3) ^d^	6.4 (4.0–10.7) ^d^
Fasting glycemia (mmol/L)	4.92 (4.55–5.1) ^a^	5.09 (4.8–5.2) ^a^	4.53 (4.39–4.55)	4.93 (4.49–5.04)	4.74 (4.48–5.05)	4.97 (4.6–5.07)	4.9 (4.5–5.06) ^d^	5.16 (4.8–5.44) ^d^
Glycemia after OGTT (mmol/L)	5.68 (5.5–8.0) ^a^	4.9 (4.7–5.7) ^a^	5.54 (4.78–5.87) ^b^	6.35 (6.14–7.27) ^b^	5.6 (5.4–6.32) ^c^	6.3 (6.1–7.2) ^c^	5.95 (5.53–7.08)	5.35 (4.92–5.89)
HOMA-IR **	2.22 (1.82–5.1)	2.6 (2.4–3.8)	2.52 (1.95–2.72)	3.4 (1.25–4.56)	2.26 (1.95–4.78)	3.4 (1.25–4.55)	2.5 (1.9–4.8) ^d^	1.51 (0.92–2.35) ^d^
T (nmol/L) **	19.1 (8.3–23.9)	9.7 (7.5–10.2)	3.78 (1.54–5.46)	1.74 (0.52–4.7)	14.4 (5.1–22.5) ^c^	1.87 (0.84–6.48) ^c^	7.08 (2.7–19.3)	1.99 (1.39–14.9)
17OHP (nmol/L) **	124.2 (20.3–420)	61.5 (4.1–70.6)	434 (258–472)	229 (20.7–410)	161 (35.2–520)	119 (20.6–329)	156 (20–410)	
Renin (ng/L) **	13.7 (4.0–29.4)	9.2 (4.5–10.2)	28.3 (7.5–57.8)	14.9 (9.1–21.8)	13.7 (5.6–31.4)	14.6 (9.0–19.8)	14.6 96.8–29.1)	
TFM (kg) ***	16.2 (13.9–18.9) ^a^	32.4 (19.7–35.7) ^a^	29.4 (18.5–32.4) ^b^	25.2 (24.6–37.4) ^b^	17.5 (14.6–23.5) ^c^	25.2 (23.5–37.4) ^c^	22.5 (16.2–27.1)	19.3 (16.8–24.7)
TFM (Z-score) ***	0.4 (0.2–0.5)	1.25 (0.1–1.3)	0.8 (−0.2–0.82) ^b^	0.3 (0.2–1.1) ^b^	0.45 (0.12–0.8)	0.3 (0.15–1.2)	0.4 (0.2–0.8) ^d^	0.05 (−0.5–0.27) ^d^
VAT (grams) ***	274 (252–359) ^a^	750 (360–800) ^a^	434 (197–450) ^b^	543 (247–566) ^b^	283 (249–387)	543 (266–662)	348 (252–501)	293 (201–358)
SAT (kg) ***	16.0 (13.7–18.5) ^a^	31.7 (19.3–32.6) ^a^	28.9 (18.3–30.5) ^b^	25.0 (24.3–36.6) ^b^	17.3 (14.4–23.1) ^c^	25.0 (23.3–36.8) ^c^	22.3 (16.0–26.6)	18.9 (16.6–18.9)
Lean body mass (kg) ***	43.3 (40.0–52.6)	56.5 (54.1–58.5)	41.1 (37.1–43.2)	41.0 (39.6–43.3)	43.3 (39.7–52.8)	43.1 (39.7–51.5)	43.2 (39.7–52.6)	44.3 (36.6–55.6)

Values are represented as median (25th–75th percentile). Bolded differences are significant. ^a^—significant difference (*p* < 0.05) was observed between the SW and SV CAH male subgroups. ^b^—significant difference was observed between the SW and SV CAH female subgroups. ^c^—significant difference was observed between the SW and SV CAH subgroups. ^d^—significant difference was observed between the CAH and control subgroups. * Adjusted for age, pubertal stage, body surface area. ** Adjusted for age, pubertal stage. *** Adjusted for age, Ht-SDS. CAH: congenital adrenal hyperplasia; SW: salt wasting; SV: simple virilizing; M: males; F: females; HC: hydrocortisone; MC: mineralocorticoid; BMI-SDS: body mass index by standard deviation score; WHR: waist and hip ratio; WHtR: waist and height ratio; BP: blood pressure; Total-C: total cholesterol; HDL-C: high-density lipoprotein cholesterol; LDL-C: low-density lipoprotein cholesterol; TG: triglycerides; OGTT: oral glucose tolerance test; HOMA-IR: homeostatic model assessment for insulin resistance; T: testosterone; 17OHP: 17-hydroxyprogesterone; TFM: total fat mass in kilograms; kg: kilograms; VAT: visceral abdominal tissue mass; SAT: subcutaneous adiposity tissue.

**Table 3:** The comparison between subgroups according to mutations in *CYP21A2* gene.

Variable	Null + Null and Null + A	Other Combinations of Mutations	*p* Value
BMI-SDS	0.48 (−0.08–1.6)	2.1 (1.6–2.8)	*p* = 0.004
Total-C	3.89 (3.23–4.2)	4.42 (4.1–5.3)	*p* = 0.046
LDL-C	2.24 (1.88–2.49)	2.75 (2.36–3.23)	*p* = 0.004
TFM (kg)	18.5 (14.9–25.0)	25.2 (22.5–37.4)	*p* = 0.041

BMI-SDS: body mass index by standard deviation score; Total-C: total cholesterol; LDL-C: low-density lipoprotein cholesterol; TFM: total fat mass in kilograms; kg: kilograms.

## Data Availability

We did not report any data.
